# The Application of Dextran Sedimentation as an Initial Step in Neutrophil Purification Promotes Their Stimulation, due to the Presence of Monocytes

**DOI:** 10.1155/2017/1254792

**Published:** 2017-10-15

**Authors:** Alex Quach, Antonio Ferrante

**Affiliations:** ^1^Department of Immunopathology, SA Pathology at the Women's and Children's Hospital, North Adelaide, SA, Australia; ^2^School of Medicine, Robinson Research Institute and School of Biological Sciences, University of Adelaide, Adelaide, SA, Australia; ^3^School of Pharmacy and Medical Sciences, Sansom Institute, University of South Australia, Adelaide, SA, Australia

## Abstract

The purification of human neutrophils for *in vitro* studies is challenging as they are easily activated through ex vivo manipulations. The technique of erythrocyte sedimentation combined with density gradient centrifugation remains widely practiced and was the subject of this study. Since in the sedimentation step the leukocytes are incubated with dextran, we have raised the likelihood that cellular activation would occur with mediator release leading to neutrophil activation. By comparing the activity of neutrophils purified from whole blood by the classical 2-step method of dextran sedimentation followed by low-density Ficoll-Hypaque (1.077 g/mL) medium, and the 1-step high-density Ficoll-Hypaque (1.114 g/mL) gradient centrifugation, we found that neutrophils from the 2-step method had a significant increase in cell surface CD11b expression and CD62L shedding and a marked increase in adhesion. Decreased random migration and chemotaxis and raised baseline oxidative burst activity were also observed. The effect was not specific to dextran, as using Ficoll for erythrocyte sedimentation replicated the elevated neutrophil adherence. Through the depletion of monocytes, lymphocytes, and platelets prior to sedimentation, we deduced that monocytes were responsible for the neutrophil activation. Our findings suggest that care needs to be exercised in choosing the method of neutrophil purification for functional studies.

## 1. Introduction

Some form of blood manipulation is required to achieve isolation of neutrophils from other blood constituents, and the challenge remains to minimise artefactual neutrophil activation. This is important as it may alter the responsiveness of neutrophils to agonists and hence undermine the experimental findings or testing for neutrophil function in diagnostic and research laboratories. There are several different methods for isolating neutrophils from human blood, including the rapid “1-step” method of centrifugation on high-density Ficoll-Hypaque (1.114 g/mL) medium [1, 2], “2-step” erythrocyte sedimentation on polysucrose-based media coupled with centrifugation on low-density Ficoll-Hypaque (1.077 g/mL) media [3, 4], and discontinuous Percoll density gradient centrifugation [5], as well as flow cytometric cell sorting [6] and immunomagnetic bead separation [7]. There are advantages and disadvantages between the uses of these techniques, but centrifugation on Ficoll-Hypaque medium and erythrocyte sedimentation techniques are the most commonly used methods.

There are variations in the separation media used but dextran-based sedimentation is typically used in conjunction with a Ficoll-Hypaque density gradient centrifugation step. In the method originally devised by Boyum [3], centrifugation on a density gradient, a concoction of a polysucrose (Ficoll) to aggregate erythrocytes and sodium diatrizoate (Hypaque) to adjust density, acts as the first step to separate out the peripheral blood mononuclear cells (PBMC), followed by dextran sedimentation to purify the neutrophils from the erythrocytes. Interestingly, many publications since also show a variation in the order of these steps, with sedimentation preceding density gradient centrifugation [5, 8–11]. This raises the possibility that monocytes under the action of dextran release mediators which activate neutrophils [12], making the separation of neutrophils by this method substandard.

To address this question, we examined the activated state of neutrophils when prepared by dextran sedimentation preceding density gradient centrifugation with those prepared by steps which removed monocytes prior to the dextran sedimentation [13–15] or with another which totally avoided the use of dextran, the 1-step density gradient centrifugation method.

In this study, we compared activity of peripheral blood neutrophils purified by 2-step dextran sedimentation preceding or following low-density Ficoll-Hypaque gradient centrifugation, along with neutrophils from the 1-step high-density Ficoll-Hypaque gradient centrifugation method. Since the neutrophils are exposed to different physical and chemical conditions, and in different chronological order, we hypothesised that neutrophils purified by each method would present with differences in their level of baseline activation that are detectable in assays of neutrophil function. In particular, we investigated this in assays of chemotaxis, respiratory/oxidase burst, bacterial killing, and adhesion.

## 2. Materials and Methods

### 2.1. Ethics Statement

Venous blood was collected from healthy adult volunteers under guidelines and approval of the Women's and Children's Health Network Human Research Ethics Committee. Written informed consent was obtained from all participants.

### 2.2. Preparation of Neutrophils

Neutrophils were prepared from heparinised peripheral blood from healthy donors by either of three methods, depending on the experiment, as previously described. In performing dextran sedimentation, every 6 mL of blood or PBMC-depleted neutrophil/erythrocyte suspension (if preceding or following low-density Ficoll-Hypaque density gradient centrifugation, resp.) was mixed with 4 mL of 5% (*w*/*v*) dextran (MW 266000, Sigma-Aldrich) in 0.9% NaCl [16]. These were incubated for 30 min at room temperature. The layer containing leukocytes or neutrophils were harvested. In performing low-density Ficoll-Hypaque gradient centrifugation, the 6 mL of blood or leukocyte-enriched plasma (if preceding or following dextran sedimentation, resp.) was layered onto low-density Ficoll-Hypaque (Ficoll-Paque Plus, 1.077 g/mL, GE Healthcare Life Sciences) and centrifuged at 400 ×g for 30 min. The neutrophil/erythrocyte or purified neutrophil pellet was resuspended and washed three times in HBSS. In performing the 1-step high-density Ficoll-Hypaque gradient centrifugation [1, 2, 17], blood was layered onto high-density Ficoll-Hypaque medium at density 1.114 g/mL and centrifuged at 600 ×g for 30 min at room temperature. The leukocytes resolved into two distinct bands, the upper containing PBMC and the lower containing neutrophils. Plasma, PBMC, and neutrophils were harvested. The cells were washed three times in HBSS. Neutrophil preparations were of 96–99% purity and were >99% viable as judged by their ability to exclude trypan blue. For some experiments with the separation media used for dextran sedimentation and Ficoll-Hypaque density gradient centrifugation, Ficoll (Ficoll 400, GE Healthcare Life Sciences) and dextran were interchanged with each other. At the conclusion of neutrophil isolation by any of the methods described above, hypotonic lysis to remove contaminating erythrocytes was performed by gently mixing 2 mL of neutrophil suspension (in HBSS) with 6 mL of sterile distilled water for 30 sec, followed by the addition of 2 mL of 3.5% NaCl (*w*/*v*).

### 2.3. Blood Reconstitution

For the reconstitution of blood with reconfiguration of various components, blood was subjected to high-density Ficoll-Hypaque gradient centrifugation with neutrophils harvested along with plasma, erythrocytes, and PBMC. In reconstituting the blood, for every 10^6^ neutrophils, 0.5 mL plasma and 0.5 mL erythrocyte pellet were for a final concentration of 10^6^/mL. For some experiments, the following components were interchanged or included: platelet-rich plasma, harvested during high-density Ficoll-Hypaque gradient centrifugation after centrifugation of blood for 10 min; platelet-depleted plasma, plasma harvested from high-density Ficoll-Hypaque gradient centrifugation as normal with the inclusion of a 3000 ×g centrifugation of the plasma for 15 min to deplete platelets; PBMC, as harvested from high-density Ficoll-Hypaque gradient centrifugation and included at 1 : 1 with the neutrophils in the same volume of blood; monocyte-depleted PBMC, using PBMC applied at 1.5 × 10^7^/cm^2^ in RPMI-1640 to a fibronectin-coated tissue culture flask (via autologous plasma for 30 min at 37°C per flask) three times—the cells were harvested and re-enumerated and checked for monocyte depletion by measuring the percentage of CD14^+^ expressing PBMC by flow cytometry using anti-CD14 PE (mouse IgG_2a_, clone M*φ*P9, BD) and anti-CD45 FITC (mouse IgG_1_, clone 2D1, BD).

### 2.4. Neutrophil Functions

Neutrophil random migration and chemotaxis was investigated using the under-agarose method as previously described [18, 19].

The neutrophil respiratory (oxidase) burst was measured by the reduction of dihydrorhodamine-123 (DHR-123) and lucigenin-dependent chemiluminescence. For DHR-123 reduction, neutrophils (5 × 10^5^) were incubated with or without 1 *μ*M DHR-123 (Sigma-Aldrich) in 500 *μ*L PBS with 0.5% BSA in FACS tubes for 15 min in a 37°C. The reaction was stopped with cold PBS supplemented with 0.5% BSA and washed once prior to acquisition on a BD FACSCanto I with excitation by the blue laser at 488 nm. The geometric mean of reduced DHR-123 was analysed using FlowJo 10.1 (FlowJo, LLC, Ashland, Oregon). For lucigenin-dependent chemiluminescence [20], neutrophils (2 × 10^5^) were incubated with 125 *μ*g lucigenin (bis-N-methylacridinium nitrate, Sigma-Aldrich) in 500 *μ*L HBSS. The chemiluminescence was measured from duplicate tubes at 37°C in a LB 953 Autolumat Plus luminometer (Berthold Technologies) over 20 min, with the data expressed as mean relative luminescence units (RLU).

Neutrophil bacterial killing was assessed by the killing of *Staphylococcus aureus* (NCTC 6571, Oxford strain) as previously described [21].

Neutrophil adherence was assessed using fibronectin-coated polypropylene microtitre plates [22]. Briefly, 96-well flat-bottom microtitre plates (Nunc) were coated with 250 *μ*L 10% autologous plasma for 30 min. Plates were washed twice and air dried before use. Neutrophils (5 × 10^5^) were allowed to adhere for 30 min at 37°C (total volume in well 200 *μ*L HBSS). Nonadherent cells were removed by inversion of the plates and the wells washed three times with 250 *μ*L HBSS and stained with 0.25% (*w*/*v*) Rose Bengal (Sigma-Aldrich). After release of the dye with 50% ethanol, the absorbance (540 nm, *A*_540_) of each well was determined using a spectrophotometric plate reader. Parallel wells were run in the absence of neutrophils. Results are expressed after subtraction of absorbance values for wells without neutrophils. Each experiment was conducted with cells from a separate individual and performed in triplicate.

Flow cytometry was used to measure the modulation of neutrophil surface adhesion receptor expression using the following fluorochrome-conjugated anti-human antibodies: anti-CD18 FITC (mouse IgG_1_, clone 6.7, BD); anti-CD11b PE (mouse IgG_2a_, clone D12, BD); anti-CD62L FITC (L-selectin, mouse IgG_1_, clone DREG56, Beckman Coulter); and anti-CD45 APC-H7 (mouse IgG_1_, clone 2D1, BD). Mouse IgG_1_ FITC (clone X40, BD) and mouse IgG_2a_ PE (clone X39, BD) served as isotype controls. Cell surface staining was performed on ice with cells in PBS with 1% foetal calf serum, incubated with antibodies for 20 min, followed by two washes in PBS with 1% foetal calf serum, prior to acquisition on a BD FACSCanto I using FlowJo 10.1 for analysis. Neutrophils were gated using CD45 APC-H7 staining versus side scatter. Median fluorescence intensity (MFI) of adhesion receptors were determined by subtraction of the MFI from the appropriate isotype control.

### 2.5. Tumour Necrosis Factor (TNF) Quantitation

The concentration of TNF was quantified in plasma, harvested from centrifuged whole blood, or the supernatant of the centrifuged leukocyte-rich plasma fraction from dextran sedimentation, using the BD™ Cytometric Bead Array Human TNF Enhanced Sensitivity Flex Set. Assays were performed in 96-well V-bottom microtitre plates (Greiner) with acquisition on a BD FACSCanto I with an attached High Throughput Sampler, using FCAP Array v3.0 (BD) for analysis. The TNF concentration was deduced from the manufacturer's standard and expressed in femtograms per mL.

### 2.6. Statistics

Graphing and statistical testing of the data was performed using Graphpad Prism 6.01 software (Graphpad Software Inc., San Diego, CA). Graphs present the data points or the mean and standard deviation unless otherwise stated. Two-tailed paired *t*-test was used except for comparison between multiple groups, where one-way analysis of variance with post hoc Dunnett's Multiple Comparisons Test was used. Statistical significance was defined as *P* < 0.05. Significance levels are indicated by asterisks; ^∗^*P* < 0.05, ^∗∗^*P* < 0.01, ^∗∗∗^*P* < 0.001, and n.s. means not significant.

## 3. Results

### 3.1. Neutrophils Purified by Dextran Sedimentation Followed by Ficoll-Hypaque Density Gradient Centrifugation Are Activated

**Figure 1:**
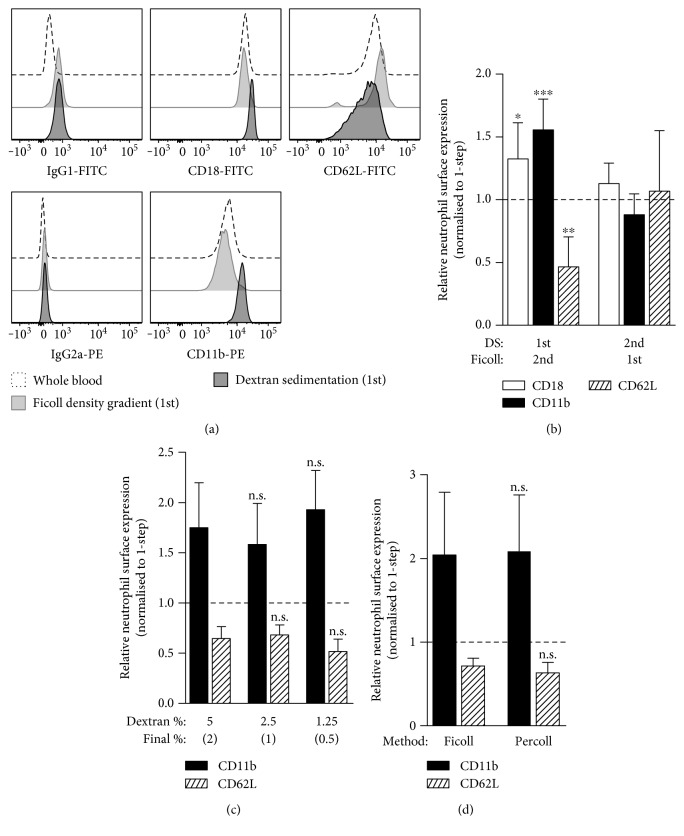
The application of dextran sedimentation prior to Ficoll-Hypaque density gradient centrifugation modulates adhesion receptor expression of neutrophils. Blood for each individual was applied to the 2-step methods, with dextran sedimentation (DS) preceding (1st) or following (2nd) Ficoll-Hypaque density gradient centrifugation, and the 1-step high-density Ficoll-Hypaque gradient centrifugation. The neutrophils were analysed for CD18, CD11b, and CD62L surface expression by flow cytometry. (a) Representative fluorescence intensity histograms of each surface neutrophil adhesion receptor molecule and respective isotype controls for purified neutrophils from each 2-step method for an individual blood sample. The histograms of unfractionated whole blood from the same sample are included for reference. (b) Relative CD18, CD11b, and CD62L surface expression between neutrophils purified by each 2-step method normalised to expression observed by neutrophils of the 1-step method (*n* = 6). (c) Relative CD11b and CD62L surface expression between neutrophils purified by different concentrations of dextran for sedimentation prior to low-density Ficoll-Hypaque gradient centrifugation normalised to expression observed by neutrophils of the 1-step method (*n* = 4). Statistical comparisons were made against a final concentration of 2% dextran. (d) Relative CD11b and CD62L surface expression between neutrophils purified by dextran sedimentation prior to low-density Ficoll-Hypaque or Percoll gradient centrifugation, normalised to expression observed by neutrophils of the 1-step method (*n* = 3).

Our investigation of purified peripheral blood human neutrophils involved three purification methods: (1) the 1-step high-density Ficoll-Hypaque gradient centrifugation (1.114 g/mL); and alternate configurations of the 2-step method where dextran sedimentation either preceded (2) or followed (3) low-density Ficoll-Hypaque gradient centrifugation (1.077 g/mL). To analyse differences in the neutrophils prepared by each of the methods, we first tested the expression levels of surface molecules associated with neutrophil adhesion, including CD18/CD11b (complement receptor 3 (CR3)) and CD62L (L-selectin) by flow cytometry. Neutrophils purified by the 1-step and the 2-step method where dextran sedimentation followed low-density Ficoll-Hypaque gradient centrifugation demonstrated comparable surface expression levels of CD18/11b and CD62L (Figures 1(a) and 1(b)). However, neutrophils purified by the 2-step method with dextran sedimentation preceding low-density Ficoll-Hypaque gradient centrifugation had significantly increased surface CD18/11b and decreased CD62L expression. Relative to the CD18/11b and CD62L expression levels of neutrophils in whole blood, the expression profile of neutrophils purified where Ficoll-Hypaque density gradient centrifugation was applied first had the closest resemblance (Figure 1(a)).

There is variation in the final concentration of dextran when mixed with blood for erythrocyte sedimentation in the literature, due to differences in the concentration of the initial dextran volume (usually 3 to 6% dextran *w*/*v* in 0.9% NaCl) and/or ratio of dextran volume to that of blood (1 : 10 to 1 : 1) [4, 5, 23–25]. Including our methodology in this study, the final concentration of dextran in the mixture with blood ranges from ~0.5 to 2%. Given that our usage (5% dextran *w*/*v* in a 1 : 1.5 dextran to blood ratio) results in a final concentration of 2%, we examined the effect of diluting the dextran to final concentrations of 1 and 0.5% (with dilutions beyond the latter not achieving a reasonable erythrocyte sedimentation, and hence not examined further) whilst maintaining the dextran to blood ratio and incubation time (30 min) for dextran sedimentation followed by low-density Ficoll-Hypaque gradient centrifugation on adhesion receptor modulation. The results showed that there was no significant difference between the dextran concentrations (Figure 1(c)).

In addition, we examined whether this adhesion receptor modulation was still evident on neutrophils when the plasma was removed prior to application of dextran, followed by density gradient centrifugation on Percoll. After centrifugation of 50 mL of blood to remove the platelet-rich plasma, we added back 5 mL of 6% dextran (*w*/*v*) to the leukocyte and erythrocyte pellet. We then topped the volume up with 50 mL 0.9% NaCl, and after effective mixing of the cell suspension, the mixture was allowed to stand for 30 min. The neutrophil/leukocyte-rich fraction was harvested and applied to a discontinuous Percoll gradient [5]. In comparison with our 2-step method with dextran sedimentation followed by low-density Ficoll-Hypaque gradient centrifugation, the usage of Percoll did not prevent the modulation of the neutrophil adhesion receptors (Figure 1(d)).

**Figure 2:**
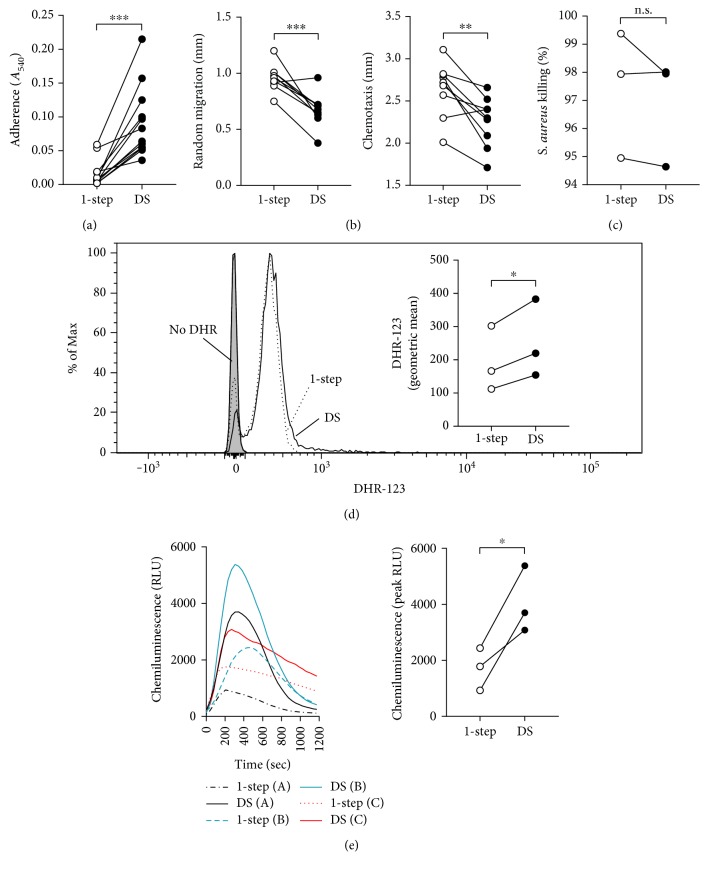
Dextran sedimentation prior to Ficoll-Hypaque density gradient centrifugation induces differences in neutrophil function compared to the 1-step method. Neutrophils were purified from blood by 1-step or 2-step with dextran sedimentation prior to low-density Ficoll-Hypaque gradient centrifugation. These cells were then applied to assays of (a) adherence (*n* = 12), (b) chemotaxis (*n* = 9), (c) *Staphylococcus aureus* killing (*n* = 3), and oxidase burst by (d) DHR-123 reduction (*n* = 3) and (e) lucigenin-dependent chemiluminescence (*n* = 3). In (d), a representative overlay histogram of reduced DHR-123 fluorescence is shown, whilst in (e), chemiluminescence kinetics plots for three individuals, A, B, and C, are presented, with a graph of the comparison between the peak RLU.

We next compared the functional profile between neutrophils purified by the 1-step method and the 2-step method with dextran sedimentation applied prior to centrifugation on Ficoll-Hypaque. The functional assays included adhesion to fibronectin-coated polypropylene, chemotaxis under agarose (with fMLF as the chemoattractant), oxidative burst by DHR-123 reduction, and bactericidal activity against *S. aureus*. The results revealed that bactericidal activity were not different between the neutrophils purified by either method (Figure 2(c)). However, neutrophils purified by dextran sedimentation followed by centrifugation on Ficoll-Hypaque showed markedly elevated adherence (>5-fold, mean = 0.091, Figure 2(a)) compared with the 1-step method (mean = 0.016), in line with the modulation of CD18/11b and CD62L described above. In addition, random migration and chemotaxis of neutrophils induced by fMLF was significantly reduced (Figure 2(b), mean random migration = 0.67 mm and chemotaxis = 2.26 mm, compared with 1-step mean random migration = 0.96 mm and chemotaxis = 2.64 mm), whilst baseline respiratory burst activity raised (mean DHR-123 MFI = 252.3 compared with 1-step MFI = 193.7 and mean peak RFU = 4053, compared with 1-step peak RFU = 1713). Overall, these results indicate that when dextran sedimentation is directly applied to blood prior to density gradient centrifugation, the neutrophils are activated.

### 3.2. Dextran and Ficoll Are Interchangeable in Erythrocyte Sedimentation for Inducing Neutrophil Activation

**Figure 3:**
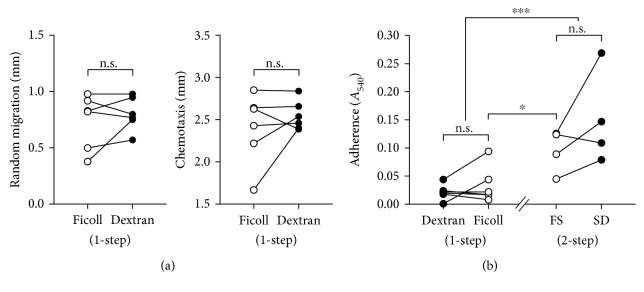
Dextran and Ficoll do not directly activate neutrophils. Blood was subjected to the dextran equivalent of 1-step high-density gradient centrifugation, and the Ficoll-equivalent of the 2-step method with erythrocyte sedimentation followed by low-density gradient centrifugation, after Ficoll 400 and dextran 266, were substituted for each other in the different purification media. Neutrophils purified by the 1-step method were assayed for chemotaxis and adherence (*n* = 6). Neutrophils purified from the 2-step sedimentation first method were assayed by adherence (*n* = 4).

Since dextran has also been used as a high molecular weight polysucrose colloidal substitute for Ficoll in the 1-step method [2, 26], it was of interest to see if neutrophil activation resulted from the dextran also in this system. Gradient media of density 1.114 g/mL was generated with Dextran-Hypaque and compared with the respective Ficoll-Hypaque for the quality of the neutrophils resolved by these systems. Examination of neutrophils purified by the 1-step method and the dextran-equivalent in the chemotaxis and adherence assays did not show any significant differences in the level of function (Figures 3(a) and 3(b)).

To further support our concept that neutrophil activation occurs when whole blood is subjected to the erythrocyte sedimentation prior to density gradient centrifugation, we subjected blood to Ficoll sedimentation followed by centrifugation on Dextran-Hypaque medium. Under these conditions, Ficoll also caused increased neutrophil adhesion (Figure 3(b)), which in relation to the results from the 1-step method revealed that the sedimentation mechanism induces neutrophil activation.

### 3.3. Neutrophil Activation Is Induced via the Presence of Mononuclear Leukocytes

**Figure 4:**
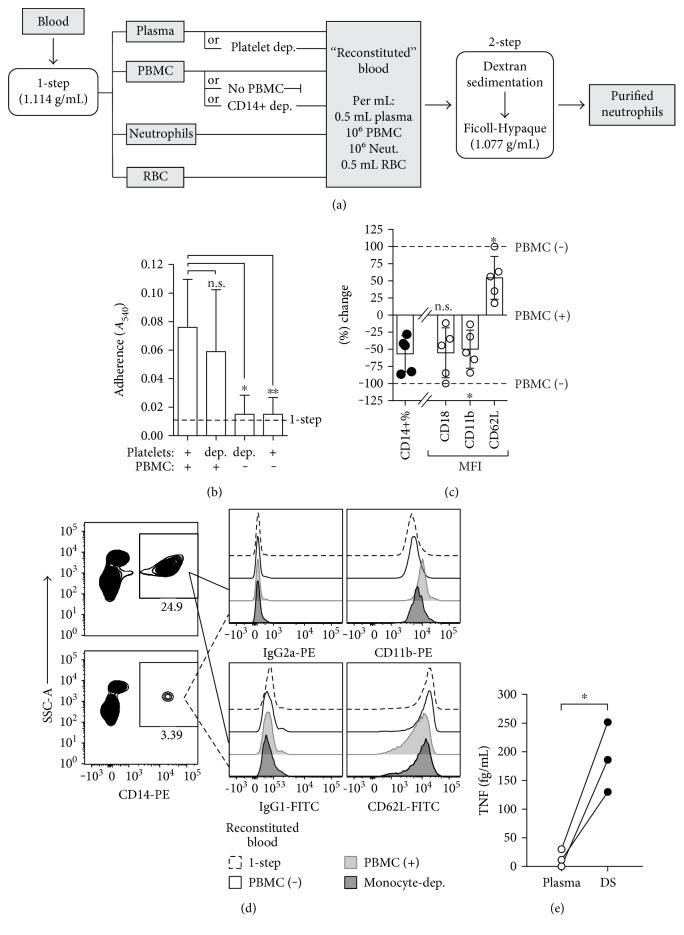
The depletion of monocytes from blood decreases adhesion modulation during dextran sedimentation. Configurations of “reconstituted” blood was applied to the 2-step method with dextran sedimentation preceding low-density Ficoll-Hypaque gradient centrifugation. (a) Schematic flow chart of the process for preparing configurations of “reconstituted” blood and the purification of neutrophils. (b) Column graph of adherence of neutrophils purified from the various configurations of blood with (+), without (−), and/or depletion (dep.) of platelets and PBMCs. (c) Graph of percentage changes from reconstituted blood with PBMC (PBMC (+)) of CD14+ monocyte % of PBMC as a result of depletion prior to application of reconstituted blood onto the 2-step method, and corresponding CD18, CD11b, and CD62L MFI of neutrophils following purification (*n* = 5). (d) Representative flow cytometry plot of CD14^+^ monocytes pre- and post-depletion from PBMCs by adhesion to fibronectin-coated polypropylene; representative histogram offset overlays of the fluorescence intensities of neutrophil surface CD11b and CD62L expression between the blood configurations. (e) TNF concentrations in the plasma extracted from blood (not applied to dextran sedimentation) and the supernatant of centrifuged leukocyte-enriched plasma immediately following dextran sedimentation (*n* = 3).

The above data led us to surmise that if the system separates the neutrophils from the PBMC, then activation of the resolved neutrophils is unlikely to occur. To test for this, we applied varying configurations of “reconstituted blood,” with or without platelets and/or PBMC, to the 2-step method of dextran sedimentation followed by centrifugation on Ficoll-Hypaque, and then compared the adherence activity of neutrophils from each blood configuration. “Reconstituted blood” was generated by applying the 1-step high-density Ficoll-Hypaque gradient centrifugation method to fractionate blood into plasma, PBMCs, neutrophils, and erythrocytes, which were then recombined with at least equal parts of plasma and erythrocytes, and 10^6^ neutrophils per mL (schematic of procedure shown in Figure 4(a)). Plasma was either platelet-rich or platelet-depleted and PBMC were excluded or added at 10^6^ per mL. We observed that the adherence of neutrophils was not significantly influenced by the amount of platelets present (Figure 4(b)). However, the level of adherence (along with the surface CD18/11b and CD62L expression profile) of the purified neutrophils in the absence of PBMCs was significantly lower, and comparable to that observed of neutrophils from the 1-step method (Figures 4(b) and 4(c)), indicating that PBMC activity during dextran sedimentation is responsible for neutrophil activation.

Given that PBMCs comprise of lymphocytes and monocytes, we sought to deduce whether the presence of either subpopulation specifically affected neutrophil activation during dextran sedimentation. Blood was fractionated and reconstituted as described above, but in this case with monocytes depleted by adhesion to fibronectin-coated culture flasks, such that the nonadherent PBMC were harvested and reincorporated into the reconstituted blood. The degree of depletion from “complete” blood (i.e., non-monocyte-depleted) was analysed by flow cytometry of CD14^+^ cells in PBMCs. We were able to achieve an average 56.7% depletion of CD14^+^ monocytes by this method (mean 15.2% to 6.1% CD14^+^ of PBMCs, Figure 4(c)). We then analysed surface CD18, CD11b, and CD62L on neutrophils following purification from the reconstituted “complete” or monocyte-depleted blood via the 2-step dextran sedimentation-first method. We found that relative to neutrophils purified from “complete” blood, the neutrophils from monocyte-depleted blood had a 55.1% and 49.9% lower surface CD18 and CD11b and a corresponding proportional 54.4% higher CD62L (Figures 4(c) and 4(d)), consistent with lower neutrophil adherence activity. The significant difference in adhesion receptor expression in correspondence with monocyte depletion clearly indicates that the monocytes exert a potent activation effect on neutrophils during dextran sedimentation, especially given that monocytes make up the minority of PBMCs.

The knowledge that TNF is a prominent cytokine released by activated monocytes that can activate and prime neutrophils prompted us to investigate if additional TNF was being produced during dextran sedimentation. We analysed the plasma of bloods prior to dextran sedimentation and the supernatants from the leukocyte-rich fraction following dextran sedimentation. We found that relative to the plasma, there was a substantial increase in TNF (mean = 189.2 fg/mL, compared with mean = 13.84 fg/mL in plasma, Figure 4(e)). Overall, to the best of our knowledge, these results establish for the first time that the presence of monocytes in blood during direct dextran sedimentation contribute to neutrophil activation.

## 4. Discussion

Dextran sedimentation remains one of the most frequently utilised techniques in neutrophil purification from human blood, typically used in a 2-step procedure with density gradient centrifugation. However, there is a lack of standardisation in its usage as various reports describe applying dextran sedimentation either prior to or following the centrifugation step (as described in several references cited in this discussion). There have also been reports that dextran sedimentation can cause activation of neutrophils [27–29]. In this study, we sought to determine how the differences in the use of these classical methodologies impact upon neutrophil activity following purification. For the first time, the data established that neutrophils become stimulated only when whole blood is exposed to dextran sedimentation prior to, but not following, Ficoll-Hypaque density gradient centrifugation. Furthermore, it was deduced that the activation was due to the sedimentation mechanism and not directly due to dextran. We also found that the activation is dependent upon the presence of monocytes during dextran sedimentation.

Surface adhesion receptor expression levels are modulated during neutrophil activation with an upregulation of CD18/11b and shedding of CD62L [30]. In this study, we showed that neutrophils purified in the 2-step method with dextran sedimentation preceding Ficoll-Hypaque density gradient centrifugation fit this expression profile of activation, when compared with those purified with dextran sedimentation following centrifugation or the 1-step high-density Ficoll-Hypaque gradient centrifugation method. The increase in CD11b expression and CD62L shedding (~0.5-fold) corresponded with a marked increase in adherence to fibronectin-coated polypropylene (>5-fold), suggesting that CR3 was activated in response to fibronectin with the level of adhesion determined by the increase in CR3 molecules. Although adherence was the most altered function in the dextran sedimentation, we also detected perturbed neutrophil random migration and chemotaxis. This is in line with evidence that these neutrophil functions have an inverse relationship where the increased adhesion to the extracellular matrix would reduce migration capacity [31, 32], in addition to our laboratory and others, previously reporting inhibition of neutrophil migration following treatment of neutrophils with an agent known to modulate adhesion, such as TNF [33–36]. Interestingly, our data showed that the deficit in migration distance against neutrophils purified by the 1-step was consistent between random migration and chemotaxis to fMLF. In addition to the effect of dextran sedimentation upon chemotaxis and adhesion, we detected an elevated baseline respiratory burst in neutrophils. These all suggest that the neutrophil preactivation can have substantial ramifications for experimental results, depending on the measurement.

Dextran is ubiquitous in that it is also utilised in the formulation of density gradients. However, Ficoll, a similarly high molecular weight polysucrose colloid, is more commonly used. Conversely, for reasons also unclear, dextran is the predominantly used agent in erythrocyte sedimentation, even though several alternatives have been used such as hydroxyethyl starch and methylcellulose [37, 38]. Our data show that whether dextran is substituted for Ficoll in density gradient centrifugation or Ficoll for dextran in erythrocyte sedimentation, the outcome of neutrophil activation following purification does not change. As such, this revealed that the mechanism of the sedimentation procedure per se caused the activation.

Various other modifications did not alter the effects of dextran on neutrophil activation. For example, the conditions where dextran sedimentation was conducted after removing the plasma, and the neutrophils further purified over Percoll gradient, led to similar activation. In addition, the activation was not related to the concentration of dextran used for the sedimentation, showing that the varied concentrations of dextran used by different laboratories lead to the same activation problem of the neutrophils.

The key mechanistic difference between density gradient centrifugation and dextran sedimentation when applied to whole blood is that the former actually fractionates plasma and PBMCs from neutrophils. Dextran sedimentation merely allows erythrocytes to sediment and permits PBMCs and neutrophils to remain in suspension with each other in a cocktail with plasma and dextran. Our elimination/depletion studies showed that monocytes exerted a significant effect upon neutrophil activation, whereas lymphocytes and platelets were not involved. It is likely that monocytes are stimulated by dextran to release mediators whilst in suspension resulting in activation of neutrophils. Interestingly, we found that the dextran sedimentation procedure leads to the release of TNF. Considering that it has been well established that the cytokine stimulates neutrophil adhesion and depresses chemotaxis, it is likely that this is a mechanism for the neutrophil activation [34, 36, 39]. While further studies will need to be conducted to identify intracellular signalling pathways induced, TNF is known to stimulate p38 MAP kinase, which is involved in the activation of neutrophils [40].

In summary, we have presented evidence that subjecting whole blood to dextran sedimentation prior to Ficoll-Hypaque density gradient centrifugation leads to neutrophil preparations that are stimulated. The preactivation was determined by measurements of increased adhesion, decreased migration, and increased oxidase burst, relative to neutrophil prepared from either the 2-step dextran sedimentation post-Ficoll-Hypaque density gradient centrifugation or the 1-step high-density Ficoll-Hypaque gradient centrifugation, which either separate the PBMC from the neutrophils or avoid the use of dextran, respectively.
